# Correction: Comparative Genomic Analysis of the Microbiome of Herbivorous Insects Reveals Eco-Environmental Adaptations: Biotechnology Applications

**DOI:** 10.1371/annotation/91a25db3-8127-42c7-baa0-ce398a2857a6

**Published:** 2013-02-05

**Authors:** Weibing Shi, Shangxian Xie, Xueyan Chen, Su Sun, Xin Zhou, Lantao Liu, Peng Gao, Nikos C. Kyrpides, En-Gyu No, Joshua S. Yuan

The word “Endosymbionts” was used incorrectly in the title, and has been replaced with “Microbiome”. The correct title is: Comparative Genomic Analysis of the Microbiome of Herbivorous Insects Reveals Eco-Environmental Adaptations: Biotechnology Applications. The correct citation is: Shi W, Xie S, Chen X, Sun S, Zhou X, et al. (2013) Comparative Genomic Analysis of the Microbiome of Herbivorous Insects Reveals Eco-Environmental Adaptations: Biotechnology Applications. PLoS Genet 9(1): e1003131. doi:10.1371/journal.pgen.1003131 

