# Supplementary figures and images for: Comparative Genomic Analysis of the Endosymbionts of Herbivorous Insects Reveals Eco-Environmental Adaptations: Biotechnology Applications
Source: PLoS Genet. 2013 Jan 10;9(1):e1003131. doi: 10.1371/journal.pgen.1003131 (PMC3542064; doi:10.1371/journal.pgen.1003131)

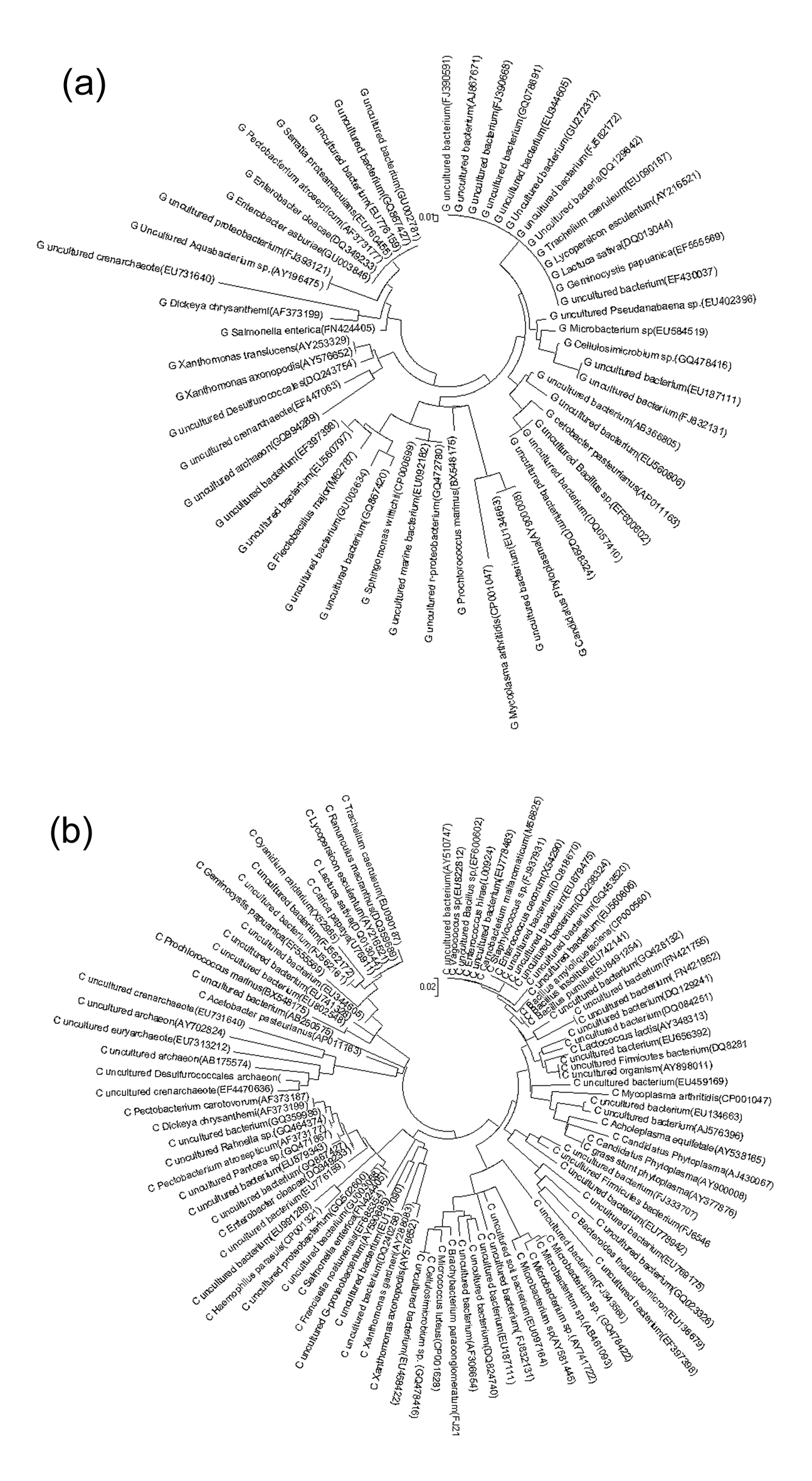

Supplement: Figure S1 — Phylogenetic tree of 16S rRNA annotated sequences. A. Grasshopper; B, cutworm. A total of 96 and 53 partial 16S rRNA sequences were extracted from cutworm and grasshopper gut microbiomes, respectively. The sequences were then aligned with the NAST aligner, and imported into an ARB database. The nearest aligned full length sequences were used for classification and phylogenetic tree construction using RAxML. Genbank accession numbers were presented in the figure. (TIF) [file pgen.1003131.s001.tif]

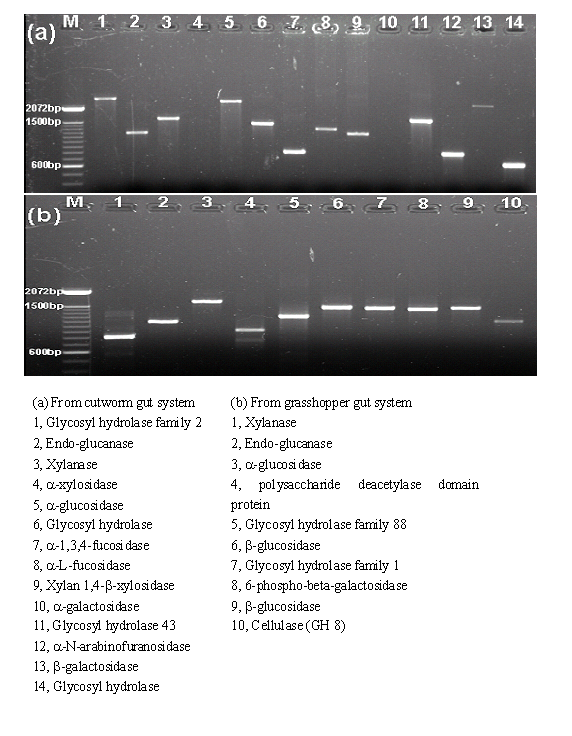

Supplement: Figure S2 — PCR amplification of cellulytic enzyme Open Reading Frames (ORFs) from the same metagenome DNA sample for sequencing library construction. (TIF) [file pgen.1003131.s002.tif]

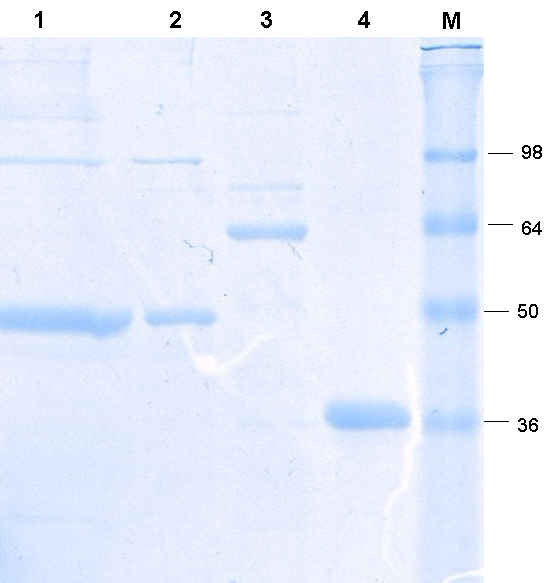

Supplement: Figure S3 — Sodium dodecyl sulfate-polyacrylamide gel electrophoresis (SDS-PAGE) analysis of purified enzymes from cutworm and grasshopper microbiomes. M: Pertained marker (Invitrogen); 1: purified endoglucanase from grasshopper (GH-EG1); 2: purified endoglucanase from cutworm (CW-EG1); 3: purified xylanase from cutworm (CW-Xyn1); 4: purified xylanase from grasshopper (GH-Xyn1). (TIF) [file pgen.1003131.s003.tif]
