# Supplementary material for: Comparative Genomic Analysis of the Endosymbionts of Herbivorous Insects Reveals Eco-Environmental Adaptations: Biotechnology Applications
Source: PLoS Genet. 2013 Jan 10;9(1):e1003131. doi: 10.1371/journal.pgen.1003131 (PMC3542064; doi:10.1371/journal.pgen.1003131)
Supplement: Table S1 — Summary of the 16S rRNA gene sequences identified from the PCR clone library of V3–V9 region for both the cutworm and grasshopper gut microbiome. (PDF) [file pgen.1003131.s005.pdf]

Shi et al., Table S1

Phylum/Class	Class	No. of sequence types in cutworm (%)	No. of sequence types in Grasshopper (%)
Proteobacteria		61 (70.1%)	86 (88.7%)
	Alpha	0	1 (1.0%)
	Beta	8 (9.2%)	0
	Gamma	52 (59.8%)	85 (87.6%)
	Unclassified	1 (1.15%)	0
Cyanobacteria	Cyanobacteria	1 (1.15%)	2 (2.1 %)
Bacteroidetes	Flavobacteria	2 (2.3%)	0
Firmicutes	Erysipelotrichi	23 (26.4%)	8 (8.2%)
Unclassified bacteria		0	1 (1.0%)
Total		87	97
