# Supplementary material for: Comparative Genomic Analysis of the Endosymbionts of Herbivorous Insects Reveals Eco-Environmental Adaptations: Biotechnology Applications
Source: PLoS Genet. 2013 Jan 10;9(1):e1003131. doi: 10.1371/journal.pgen.1003131 (PMC3542064; doi:10.1371/journal.pgen.1003131)
Supplement: Table S2 — Enriched or under-represented KEGG pathway categories in grasshopper and cutworm gut microbiome as compared to those of termite gut. (PDF) [file pgen.1003131.s006.pdf]

Shi et al., Table S2

KEGG category (Pathway)	Grasshopper			Cutworm		
	Hits	D-rank*	*p*-value	Hits	D-rank*	*p*-value
**Carbohydrate Metabolism**						
Glycolysis / Gluconeogenesis	73	1.4	0.08	121	2.72	<0.01
Fructose and mannose metabolism	69	1.13	0.13	107	2.2	<0.05
Galactose metabolism	64	1.49	0.07	106	2.89	<0.01
Ascorbate and aldarate metabolism	66	1.38	0.08	94	2.68	<0.01
Starch and sucrose metabolism	78	1.39	0.08	134	3.17	<0.01
Aminosugars metabolism	67	1.41	0.08	94	2.75	<0.01
**Energy Metabolism**						
Oxidative phosphorylation	33	-1.71	<0.05	16	-0.87	0.19
Photosynthesis	9	-3.28	<0.01	12	-2.01	<0.05
**Xenobiotics Biodegradation and Metabolism**						
Fluorobenzoate degradation	2	0	<0.01	1	0	<0.01
**Metabolism of Other Amino Acids**						
Cyanoamino acid metabolism	10	0.66	0.25	24	2.24	<0.05
**Glycan Biosynthesis and Metabolism**						
O-Glycan biosynthesis	1	0	<0.01	1	0	<0.01
**Biosynthesis of Secondary Metabolites**						
Phenylpropanoid biosynthesis	10	0.51	0.30	24	1.74	<0.05
Polyketide sugar unit biosynthesis	3	0	0.50	20	2.11	<0.05
Biosynthesis of siderophore group nonribosomal peptides	23	1.68	<0.05	5	0	0.50
Biosynthesis of type II polyketide backbone	17	2.74	<0.01	2	0	0.50
**Cell Motility**						
Flagellar assembly	7	-5.68	<0.01	12	-3.48	<0.01
**Membrane Transport**						
Phosphotransferase system (PTS)	46	6.4	<0.01	82	12.45	<0.01
**Sorting and Degradation**						
Type III secretion system	7	-5.68	<0.01	12	-3.48	<0.01

* D-ranks were derived for the grasshopper or cutworm gut microbiome against termite gut microbiome in KEGG. All pathways in the table were the enriched or under-represented ones when compared to termite gut microbiomes ($P < 0.05$). The D-ranks were a measurement of relative gene content based on the number of independent hits in each pathway; See http://gordonlab.wustl.edu/supplemental/Gill/ for each of these maps.
