# Supplementary material for: Comparative Genomic Analysis of the Endosymbionts of Herbivorous Insects Reveals Eco-Environmental Adaptations: Biotechnology Applications
Source: PLoS Genet. 2013 Jan 10;9(1):e1003131. doi: 10.1371/journal.pgen.1003131 (PMC3542064; doi:10.1371/journal.pgen.1003131)
Supplement: Table S3 — Comparison of Glycosyl Hydrolase (GH), Carbohydrate Binding Modules (CBM), and Glycosyl Transferase (GT) domain counts in grasshopper (GH), cutworm (CW), and termite (TM). GH stands for grasshopper, CW stands for cutworm, and TM stands for termite. (PDF) [file pgen.1003131.s007.pdf]

Shi et al., Table S3

Pfam ID	Pfam HMM name	Known activities	GH	CW	TM
**Glycoside hydrolase catalytic domains**					
pfam00232	GH_1	β-Glucosidase, β-galactosidase, β-mannosidase, others	34	181	27
pfam00703	GH_2	β-galactosidase, β-mannosidase, others	1	9	33
pfam02836	GH_2_C	β-galactosidase, β-mannosidase, others	1	13	40
pfam02837	GH_2_N	Sugar-binding domain	1	10	48
pfam00933	GH_3	β-glucosidase, xylan1,4-β-xylosidase, β-N-acetylhexosaminidase, others	4	22	109
pfam01915	GH_3_C	C terminal domain (glucan-binding)	2	22	61
pfam02056	GH_4	β-Glucosidase, α-galactosidase, others	5	18	20
pfam11975	GH_4C	C-terminal domain	5	16	26
pfam00150	GH_5	Cellulase	1	1	125
pfam01270	GH_8	Cellulase, Chitosanase, Endoglucanase Y, others	1	0	21
pfam00759	GH_9	Endoglucanase, cellobiohydrolase	0	0	44
pfam00331	GH_10	Xylanase, β-1,3-endoxylanase	0	0	101
pfam00457	GH_11	Xylanase	0	0	19
pfam00128	Alpha_amylase	Alpha amylase, catalytic domain	18	18	119
pfam02806	Alpha_amylase_N	Alpha amylase, N-terminal ig-like domain	2	2	1
pfam02903	Alpha_amylase_C	Alpha amylase, C-terminal all-beta domain	2	2	8
pfam00723	GH_15	Glucoamylase, glucodextranase, α,α-trehalase	1	0	0
pfam00722	GH_16	β-1,3(4)-Endoglucanase, others	0	0	6
pfam00704	GH_18	Chitinase, endo-β-*N*-acetylglucosaminidase, others	4	2	28
pfam00182	GH_19	Chitinase	1	0	1
pfam00728	GH_20	β-Hexosaminidase, lacto-*N*-biosidase	2	5	18
pfam02838	GH_20b	*N*-acetyl-beta-hexosaminidase, domain 2	1	0	5
pfam01464	SLT	Soluble lytic murein transglycosylase and related regulatory proteins	8	2	63
pfam01183	GH_25	Lyzozyme M1 (1,4-β-*N*-acetylmuramidase)	1	2	4
pfam02156	GH_26	β-mannanase, β-1,3-xylanase	0	0	20
pfam02065	Melibiase	α-galactosidase, Melibiase	0	12	13
pfam00295	GH_28	Endopolygalacturonase, others	2	8	15
pfam02055	GH_30	glucosylceramidase	0	0	13
pfam01055	GH_31	α-glucosidase, others	12	18	37
pfam08244	GH_32C	C-terminal domain; β-fructosidases	0	2	0
pfam00251	GH_32N	N-terminal domain, β-fructosidases, β-xylosidase	0	5	2
pfam01301	GH_35	β-galactosidase	0	10	6
pfam01074	GH_38	α-mannosidase	2	11	18
pfam07748	GH_38C	C-terminal domain	2	5	26
pfam01229	GH_39	β-xylosidase, α-L-iduronidase	0	3	15
pfam02449	GH_42	β-galactosidase	0	29	39
pfam08533	GH_42C	C-terminal domain	1	5	9

pfam08532	GH_42M	β-galactosidase trimerisation domain	0	18	27
pfam04616	GH_43	β-xylosidase, α-L-arabinofuranosidase, arabinanase, and xylanase	4	32	63
pfam02015	GH_45	Endoglucanase	0	0	6
pfam03512	GH_52	β-xylosidase	0	0	3
pfam07745	GH_53	Arabinogalactan, endo-1,4-β-galactosidase	1	3	21
pfam03065	GH_57	α-amylase, α-mannosidase, others	0	0	52
pfam03633	GH_65C	C-terminal domain, Trehalose and maltose hydrolases	0	3	3
pfam03632	GH_65m	Central catalytic domain, Trehalose and maltose hydrolases	1	19	11
pfam03636	GH_65N	N-terminal domain, Trehalose and maltose hydrolases	1	12	4
pfam07477	GH_67C	C-terminal domain, α-glucuronidase	0	2	12
pfam07488	GH_67M	Middle domain, α-glucuronidase	0	1	14
pfam03648	GH_67N	N-terminal domain, α-glucuronidase	0	0	6
pfam03663	GH_76	α-1,6-mannanases	0	1	0
pfam02446	GH_77	4-alpha-glucanotransferase, amylomaltase	5	2	34
pfam03662	GH_79N	N-terminal domain	0	0	1
pfam03644	GH_85	Endo-β-N-acetylglucosaminidase D	0	3	0
pfam07470	GH_88	Unsaturated glucuronyl hydrolase	4	14	19
pfam07971	GH_92	α-1,2-mannosidases	0	7	3
pfam10566	GH_97	α-glucosidase, α-galactosidase	0	0	1
pfam08306	GH_98M	Endo-β-glactosidase	0	0	1
Total of GH family enzymes			130	550	1421
**Carbohydrate Binding Modules (CBM)**					
pfam02018	CBM_4_9	Amorphous cellulose-, xylan- and glucan-binding domain	0	0	9
pfam02839	CBM_5_12	C-terminal cellulose-binding domain of endoglucanase	2	0	0
pfam02922	CBM_48	Isoamylase N-terminal domain	8	6	22
pfam03422	CBM_6	Amorphous cellulose- and xylan-binding domain	0	0	18
pfam03425	CBM_11	Glucan-binding domain	0	0	12
pfam06204	CBM_X	Putative carbohydrate binding domain	0	0	47
pfam08305	NPCBM	NPCBM/NEW2 domain	0	0	4
pfam09212	CBM27	β-1, 4-mannooligosaccharides, carob galactomannan, and konjac glucomannan-binding domain	0	0	1
Total of CBM			10	6	113
**Glycosyl Transferases**					
pfam00852	GT_10	Galactoside, 3(4)-L-fucosyltransferase, and galactoside 3-fucosyltransferase	0	0	1
pfam00982	GT_20	α, α-trehalose-phosphate synthase [UDP-forming]	2	0	0
pfam01075	GT_9	lipopolysaccharide N-acetylglucosaminyltransferase, heptosyltransferase	8	1	4
pfam01501	GT_8	lipopolysaccharide galactosyltransferase, lipopolysaccharide glucosyltransferase, others	1	2	6
pfam01755	GT_25	lipopolysaccharide biosynthesis protein	1	0	0
pfam03033	GT_28	β-galactosyltransferase, β-glucosyltransferase, β-N-acetylglucosamine transferase	2	3	20

pfam06165	GT_36	cellobiose phosphorylase, cellodextrin phosphorylase, and chitobiose phosphorylase	0	0	59
pfam08323	GT_5	glycogen/starch synthases, ADP-glucose type	2	4	32
Total of GT enzymes			16	10	122
