# Supplementary material for: Comparative Genomic Analysis of the Endosymbionts of Herbivorous Insects Reveals Eco-Environmental Adaptations: Biotechnology Applications
Source: PLoS Genet. 2013 Jan 10;9(1):e1003131. doi: 10.1371/journal.pgen.1003131 (PMC3542064; doi:10.1371/journal.pgen.1003131)
Supplement: Table S4 — Distribution of genes belonging to the phosphotransferase system (PTS) in the grasshopper (GH), cutworm (CW), and termite (TM). (PDF) [file pgen.1003131.s008.pdf]

Shi et al., Table S4

COG	PTS name	No. of Hits		
		GH	CW	TM
COG1263	Phosphotransferase system IIC components, glucose/maltose/N-acetylglucosamine-specific	14	53	1
COG1264	Phosphotransferase system IIB components	7	27	0
COG1299	Phosphotransferase system, fructose-specific IIC component	10	20	0
COG1440	Phosphotransferase system cellobiose-specific component IIB	6	17	0
COG1445	Phosphotransferase system fructose-specific component IIB	4	9	0
COG1447	Phosphotransferase system cellobiose-specific component IIA	7	18	0
COG1455	Phosphotransferase system cellobiose-specific component IIC	30	134	0
COG1762	Phosphotransferase system mannitol/fructose-specific IIA domain (Ntr-type)	13	24	60
COG1925	Phosphotransferase system, HPr-related proteins	3	2	18
COG2190	Phosphotransferase system IIA components	3	49	0
COG2213	Phosphotransferase system, mannitol-specific IIBC component	2	15	0
COG2893	Phosphotransferase system, mannose/fructose-specific component IIA	4	9	0
COG3414	Phosphotransferase system, galactitol-specific IIB component	6	10	1
COG3444	Phosphotransferase system, mannose/fructose/N-acetylgalactosamine-specific component IIB	5	21	0
COG3715	Phosphotransferase system, mannose/fructose/N-acetylgalactosamine-specific component IIC	7	19	0
COG3716	Phosphotransferase system, mannose/fructose/N-acetylgalactosamine-specific component IID	7	25	1
COG3730	Phosphotransferase system sorbitol-specific component IIC	0	1	0
COG3731	Phosphotransferase system sorbitol-specific component IIA	0	1	0
COG3732	Phosphotransferase system sorbitol-specific component IIBC	0	2	0
COG3775	Phosphotransferase system, galactitol-specific IIC component	0	6	0
COG3925	N-terminal domain of the phosphotransferase system fructose-specific component IIB	1	0	0
COG4668	Mannitol/fructose-specific phosphotransferase system, IIA domain	1	2	0
Total		130	464	81
