# Supplementary material for: Comparative Genomic Analysis of the Endosymbionts of Herbivorous Insects Reveals Eco-Environmental Adaptations: Biotechnology Applications
Source: PLoS Genet. 2013 Jan 10;9(1):e1003131. doi: 10.1371/journal.pgen.1003131 (PMC3542064; doi:10.1371/journal.pgen.1003131)
Supplement: Table S5 — Comparison of grasshopper (G) and cutworm (C) gut microbiome with termite (T) gut microbiome showed the enrichment of energy production and conversion COGs. (PDF) [file pgen.1003131.s009.pdf]

Shi et al., Table S5

COG	Annotation	Grasshopper		Cutworm		D-score G/C
		Hits	*D-score G/T	Hits	*D-score C/T	
COG0039	Malate/lactate dehydrogenases	3	/	9	4.10	/
COG0055	F0F1-type ATP synthase, beta subunit	31	5.42	23	2.47	/
COG0056	F0F1-type ATP synthase, alpha subunit	36	5.91	25	2.38	/
COG0074	Succinyl-CoA synthetase, alpha subunit	5	2.88	2	/	/
COG0356	F0F1-type ATP synthase, subunit a	8	2.86	4	/	/
COG0377	NADH:ubiquinone oxidoreductase 20 kD subunit and related Fe-S oxidoreductases	10	4.27	5	/	/
COG0437	Fe-S-cluster-containing hydrogenase components 1	10	4.27	2	/	/
COG0538	Isocitrate dehydrogenases	3		10	2.94	/
COG0584	Glycerophosphoryl diester phosphodiesterase	5	2.61	6	2.56	/
COG0649	NADH:ubiquinone oxidoreductase 49 kD subunit 7	26	8.32	4	/	/
COG0667	Predicted oxidoreductases (related to aryl-alcohol dehydrogenases)	16	4.99	21	5.37	/
COG0838	NADH:ubiquinone oxidoreductase subunit 3 (chain A)	7	2.96	2	/	/
COG0852	NADH:ubiquinone oxidoreductase 27 kD subunit	7	2.58	1	/	/
COG1005	NADH:ubiquinone oxidoreductase subunit 1 (chain H)	19	6.58	8	2.04	/
COG1008	NADH:ubiquinone oxidoreductase subunit 4 (chain M)	23	7.89	9	2.58	3.24
COG1009	NADH:ubiquinone oxidoreductase subunit 5 (chain L)/Multisubunit Na+/H+ antiporter, MnhA subunit	29	9.79	13	4.33	3.34
COG1012	NAD-dependent aldehyde dehydrogenases	36	5.91	14	/	4.07
COG1013	Pyruvate:ferredoxin oxidoreductase and related 2-oxoacid:ferredoxin oxidoreductases, beta subunit	1	-3.96	7	-3.23	/
COG1038	Pyruvate carboxylase	0	/	24	8.15	/
COG1042	Acyl-CoA synthetase (NDP forming)	6	3.42	0	/	/
COG1143	Formate hydrogenlyase subunit 6/NADH:ubiquinone oxidoreductase 23 kD subunit (chain I)	13	3.09	7	/	/
COG1301	Na+/H+-dicarboxylate symporters	14	4.05	3	/	/
COG1882	Pyruvate-formate lyase	19	5.19	15	3.07	/
COG1894	NADH:ubiquinone oxidoreductase, NADH-binding (51 kD) subunit	7	-2.81	3	-4.44	/
COG4232	Thiol:disulfide interchange protein	8	3.03	0	-1.67	/

* D-score with the number means this COG in grasshopper or cutworm gut microbiome is significantly enrichment or under-represented compared to termite gut microbiome (*P*<0.05)
