# Supplementary material for: Comparative Genomic Analysis of the Endosymbionts of Herbivorous Insects Reveals Eco-Environmental Adaptations: Biotechnology Applications
Source: PLoS Genet. 2013 Jan 10;9(1):e1003131. doi: 10.1371/journal.pgen.1003131 (PMC3542064; doi:10.1371/journal.pgen.1003131)
Supplement: Table S6 — Comparison of grasshopper (GH) and cutworm (CW) gut microbiome with termite (TM) gut microbiome showed the enrichment for nitrogen metabolism KEGGs. (PDF) [file pgen.1003131.s010.pdf]

Function ID	Annotation	No. of Hits		
		GH	CW	TM
EC:1.18.6.1	Nitrogenase.	0	0	22
EC:1.4.1.13	Glutamate synthase (NADPH).	4	0	52
EC:1.4.1.14	Glutamate synthase (NADH).	4	0	9
EC:1.4.1.4	Glutamate dehydrogenase (NADP(+)).	0	0	3
EC:1.4.7.1	Glutamate synthase (ferredoxin).	1	0	0
EC:1.7.1.4	Nitrite reductase (NAD(P)H).	1	0	0
EC:1.7.99.4	Nitrate reductase.	3	0	0
EC:2.1.2.10	Aminomethyltransferase.	1	0	1
EC:2.7.2.2	Carbamate kinase.	1	0	8
EC:3.5.1.1	Asparaginase.	0	0	2
EC:4.1.99.1	Tryptophanase.	0	0	2
EC:4.2.1.1	Carbonate dehydratase.	4	0	4
EC:4.3.1.3	Histidine ammonia-lyase.	0	0	2
EC:4.4.1.8	Cystathionine beta-lyase.	0	0	1
EC:6.3.1.1	Aspartate--ammonia ligase.	0	0	1
EC:6.3.1.2	Glutamate--ammonia ligase.	1	0	0
EC:6.3.1.5	NAD(+) synthase.	0	0	4
EC:6.3.5.4	Asparagine synthase (glutamine-hydrolyzing).	3	1	0
