# Supplementary material for: Comparative Genomic Analysis of the Endosymbionts of Herbivorous Insects Reveals Eco-Environmental Adaptations: Biotechnology Applications
Source: PLoS Genet. 2013 Jan 10;9(1):e1003131. doi: 10.1371/journal.pgen.1003131 (PMC3542064; doi:10.1371/journal.pgen.1003131)
Supplement: Table S7 — Enrichment of defense-related genes in gut microbiomes of grasshopper (GH), cutworm (CW), and termite (TM). (PDF) [file pgen.1003131.s011.pdf]

Shi et al., Table S7

Function ID	Name	GH	CW	TM
COG0286	Type I restriction-modification system methyltransferase subunit	2	7	0
COG0534	Na+-driven multidrug efflux pump	4	3	0
COG0577	ABC-type antimicrobial peptide transport system, permease component	2	1	0
COG0610	Type I site-specific restriction-modification system, R (restriction) subunit and related helicases	1	4	0
COG0732	Restriction endonuclease S subunits	1	4	0
COG0841	Cation/multidrug efflux pump	32	0	0
COG0842	ABC-type multidrug transport system, permease component	2	0	0
COG1002	Type II restriction enzyme, methylase subunits	0	0	0
COG1131	ABC-type multidrug transport system, ATPase component	21	73	1
COG1132	ABC-type multidrug transport system, ATPase and permease components	28	144	0
COG1136	ABC-type antimicrobial peptide transport system, ATPase component	12	66	0
COG1401	GTPase subunit of restriction endonuclease	1	2	0
COG1403	Restriction endonuclease	0	1	0
COG1566	Multidrug resistance efflux pump	20	0	0
COG1619	Uncharacterized proteins, homologs of microcin C7 resistance protein MccF	4	0	0
COG1680	Beta-lactamase class C and other penicillin binding proteins	4	3	0
COG1715	Restriction endonuclease	1	0	0
COG1787	Predicted endonuclease distantly related to archaeal Holliday junction resolvase and Mrr-like restriction enzymes	0	2	0
COG1968	Uncharacterized bacitracin resistance protein	3	5	0
COG2274	ABC-type bacteriocin/lantibiotic exporters, contain an N-terminal double-glycine peptidase domain	14	21	0
COG2348	Uncharacterized protein involved in methicillin resistance	1	1	0
COG2367	Beta-lactamase class A	1	0	0
COG2602	Beta-lactamase class D	0	0	0
COG2720	Uncharacterized vancomycin resistance protein	0	0	0
COG2746	Aminoglycoside N3'-acetyltransferase	1	2	0
COG2810	Predicted type IV restriction endonuclease	0	0	0
COG3023	Negative regulator of beta-lactamase expression	5	1	0
COG3183	Predicted restriction endonuclease	2	1	0
COG3440	Predicted restriction endonuclease	2	1	0
COG3510	Cephalosporin hydroxylase	0	0	0
COG3570	Streptomycin 6-kinase	0	0	0
COG3587	Restriction endonuclease	0	0	1
COG3725	Membrane protein required for beta-lactamase induction	1	0	0
COG3896	Chloramphenicol 3-O-phosphotransferase	0	0	0
COG4096	Type I site-specific restriction-modification system, R (restriction) subunit and related helicases	1	0	2

COG4167	ABC-type antimicrobial peptide transport system, ATPase component	2	0	0
COG4168	ABC-type antimicrobial peptide transport system, permease component	3	0	0
COG4170	ABC-type antimicrobial peptide transport system, ATPase component	1	0	0
COG4171	ABC-type antimicrobial peptide transport system, permease component	1	0	0
COG4257	Streptogramin lyase	0	0	0
COG4268	McrBC 5-methylcytosine restriction system component	1	1	0
COG4403	Lantibiotic modifying enzyme	0	1	0
COG4452	Inner membrane protein involved in colicin E2 resistance	1	0	1
COG4767	Glycopeptide antibiotics resistance protein	0	1	0
COG4823	Abortive infection bacteriophage resistance protein	0	4	0
COG4845	Chloramphenicol O-acetyltransferase	0	0	0
